# Microsatellite polymorphism within *pfcrt *provides evidence of continuing evolution of chloroquine-resistant alleles in Papua New Guinea

**DOI:** 10.1186/1475-2875-6-34

**Published:** 2007-03-21

**Authors:** Jeana T DaRe, Rajeev K Mehlotra, Pascal Michon, Ivo Mueller, John Reeder, Yagya D Sharma, Mark Stoneking, Peter A Zimmerman

**Affiliations:** 1 Case Western Reserve University, Wolstein Research Building #4125, 2103 Cornell Road, Cleveland, OH 44106, USA; 2 Papua New Guinea Institute of Medical Research, P.O. Box 60, Goroka, Papua New Guinea; 3 Burnet Institute for Medical Research and Public Health, P.O. Box 2284, Melbourne, VIC 2001, Australia; 4 Department of Biotechnology, All India Institute of Medical Sciences, Ansari Nagar, New Delhi-110029, India; 5 Max Planck Institute for Evolutionary Anthropology, Deutscher Platz 6, D-04103, Leipzig, Germany

## Abstract

**Background:**

Polymorphism in the *pfcrt *gene underlies *Plasmodium falciparum *chloroquine resistance (CQR), as sensitive strains consistently carry lysine (K), while CQR strains carry threonine (T) at the codon 76. Previous studies have shown that microsatellite (MS) haplotype variation can be used to study the evolution of CQR polymorphism and to characterize intra- and inter-population dispersal of CQR in Papua New Guinea (PNG).

**Methods:**

Here, following identification of new polymorphic MS in introns 2 and 3 within the *pfcrt *gene (msint2 and msint3, respectively), locus-by-locus and haplotype heterozygosity (*H*) analyses were performed to determine the distribution of this intronic polymorphism among *pfcrt *chloroquine-sensitive and CQR alleles.

**Results:**

For MS flanking the *pfcrt *CQR allele, *H *ranged from 0.07 (B5M77, -18 kb) to 0.094 (9B12, +2 kb) suggesting that CQ selection pressure was responsible for strong homogenisation of this gene locus. In a survey of 206 *pfcrt*-SVMNT allele-containing field samples from malaria-endemic regions of PNG, *H *for msint2 was 0.201. This observation suggests that *pfcrt *msint2 exhibits a higher level of diversity than what is expected from the analyses of *pfcrt *flanking MS. Further analyses showed that one of the three haplotypes present in the early 1980's samples has become the predominant haplotype (frequency = 0.901) in CQR parasite populations collected after 1995 from three PNG sites, when CQR had spread throughout malaria-endemic regions of PNG. Apparent localized diversification of *pfcrt *haplotypes at each site was also observed among samples collected after 1995, where minor CQR-associated haplotypes were found to be unique to each site.

**Conclusion:**

In this study, a higher level of diversity at MS loci within the *pfcrt *gene was observed when compared with the level of diversity at *pfcrt *flanking MS. While *pfcrt *(K76T) and its immediate flanking region indicate homogenisation in PNG CQR parasite populations, *pfcrt *intronic MS variation provides evidence that the locus is still evolving. Further studies are needed to determine whether these intronic MS introduce the underlying genetic mechanisms that may generate *pfcrt *allelic diversity.

## Background

*Plasmodium falciparum *chloroquine resistance (CQR) was first reported in Southeast Asia and South America during the late 1950's [1]. Since then, CQR parasites have spread worldwide, with corresponding increases in malaria morbidity and mortality [2]. CQR in Papua New Guinea (PNG) was first reported in 1976 [3,4]. By the early 1980's, *in vivo *studies indicated that CQR *P. falciparum *was present in ~50% of the children, while *in vitro *assays revealed that ~80% of the isolates were CQR [5,6]. Recent molecular studies have shown that CQR-associated alleles had spread throughout PNG by the mid 1980's [7,8], and based on widespread chloroquine (CQ) treatment failure by the late 1990's [7,9,10], the PNG Ministry of Health changed its guidelines for malaria treatment in 2000 from CQ alone to CQ + sulphadoxine-pyrimethamine.

Genetic polymorphism associated with the CQR phenotype in *P. falciparum *has been identified in the *P. falciparum *chloroquine resistance transporter (*pfcrt*) gene, located on chromosome 7 [11-13]. The amino acid substitution at *pfcrt *codon 76 (K→T) has been shown to have the strongest association with the CQR phenotype, which can be both reversible and irreversible by verapamil [7,11,14-20]. The *pfcrt *gene encodes an integral membrane protein, which is localized to the parasite digestive vacuole [11] where haem molecules released during haemoglobin digestion are detoxified by the formation of haemozoin, also known as malaria pigment; CQ is suggested to interfere with this process [21,22]. *P. falciparum *CQR is suggested to involve mechanisms whereby pH sensitive physiologic processes inhibit formation of toxic CQ:haematin complexes in favor of haemozoin [22], or CQ efflux reduces drug concentration to the levels that are no longer parasiticidal [23-25].

In addition to *pfcrt*, *P. falciparum *multidrug resistance (*pfmdr1*, chromosome 5) and nine other putative transporter genes have been implicated in CQR [7,26-28]. Polymorphisms in *pfmdr1 *play a modulatory role in CQR [29], while those in only one of the other nine transporter genes (*G7*, encoding an ATP-binding cassette transporter [PlasmoDB identifier: PF13-0271]) exhibit significant association with response to the antimalarial drug artesunate [28].

To understand further the population genetics of CQR *P. falciparum *in PNG, samples collected from six different provinces in the early 1980's and after 1995, in both community and clinical settings, were analysed. *P. falciparum *samples were classified by genotyping *pfcrt *codons 72–76 (CVMNK represents chloroquine-sensitive [CQS] allele, SVMNT and CVIET CQR alleles), and were further analysed for *pfcrt *single nucleotide polymorphisms (SNPs) at codons 220 (A→S), 271 (Q→E), 326 (N→D), 356 (I→L), and 371 (R→I) [11,30], as well as 152 (T→A) and 163 (S→R), which have been recently implicated in parasite's resistance to amantadine and modification of verapamil-reversible CQR phenotype [30]. A series of four microsatellite (MS) loci and the *cg2 *ω repeat region flanking the *pfcrt *locus (-18 kb upstream to +19 kb downstream), and recently discovered MS loci within the *pfcrt *gene [31] were analysed to investigate the diversity and distribution of CQS and CQR alleles. MS markers occurring at regular 2–3 kb intervals in the *P. falciparum *genome [32] have been recently used to analyse both intra- and inter-population relationships among drug-resistant *P. falciparum *strains, based on the extent of linkage disequilibrium (LD) and its decay rates [33-35]. In this study, these same approaches were used to provide new insight into the dispersal and continuing evolution of CQR *P. falciparum *strains in PNG.

## Methods

### Collection of samples

Samples were collected from both placental tissues and whole blood. Seven placental tissues samples were obtained from pregnant women living in the Eastern Highlands (n = 1), East Sepik (n = 3), Manus (n = 1), Milne Bay (n = 1), and Morobe (n = 1) provinces of PNG between 1982 and 1984 [36]. Clinical data were not available for these placental samples. Peripheral blood samples were collected into K^+^-EDTA containing Vacutainer tubes from individuals living in three malaria-holoendemic regions of PNG [37,38]. These samples were collected from the Dreikikir region of East Sepik Province (ESP) in 1996 (n = 31), the Liksul region of Madang Province in 1996 (n = 22), and the Wosera region of ESP. The Wosera samples were collected in 1998 (n = 65) and 2002 (n = 182) during community surveys, and between 2001 and 2003 (n = 980) from symptomatic (e.g., fever, parasitaemia) patients at the local health centers. Study protocols were reviewed and approved by the Medical Research Advisory Committee, Department of Health PNG, and the University Hospitals of Cleveland Institutional Review Board.

### Genomic DNA preparation and parasite reference strains

DNA was extracted from the placental tissue samples by a standard phenol/chloroform extraction method [36], and from the whole blood (200 μl) using the QIAamp 96 Blood Kit (QIAGEN, Valencia, CA). For MS allele analyses, genomic DNA preparations of six *P. falciparum *laboratory-adapted strains (HB3, 3D7, Dd2, K1, 7G8, and PNG1917) were used as references.

### PCR amplification and genotyping of *pfcrt *codons

Polymerase Chain Reactions (PCR) to amplify *pfcrt *codons 72–76, 220, 271, 326, 356, and 371 were performed as previously described [7]. PCR to amplify the codons 152 and 163 were performed using the primers and conditions described in Additional File 1. PCR products were genotyped for the above polymorphic codons using ligase detection reaction-fluorescent microsphere assay (LDR-FMA) as recently described [39-41]. The allele-specific probes used in LDR-FMA are provided in Additional File 2.

### PCR amplification and genotyping of *cg2 *ω repeat region and MS loci

Amplification and genotyping of MS flanking *pfcrt *(B5M77, 2E10, 9B12, and 2H4), *cg2 *ω repeat region, and a putatively neutral locus PfPK2 (chromosome 12) were performed using semi-nested PCR strategies as previously described [8,42]. *Pfcrt *intronic MS msint1, also known as B5M47 [13], and the newly discovered intronic MS msint2 and msint3 were amplified using nested PCR strategies; primer sequences and conditions for these amplifications are provided in Additional File 1. For each MS, one of the nest-2 amplification primers was 5' end-labeled with Cy5. PCR products were mixed 3:1 (vol/vol) with denaturing loading dye buffer (formamide 10 ml, bromophenol blue 10 mg, 0.5 M EDTA [pH 8.0] 200 μl) and denatured at 95°C for 10 min. Denatured products were run on a 6% denaturing polyacrylamide gel (6.3 M urea/32% formamide) for 3 h in a Gibco BRL sequencing apparatus (model S2, Gibco BRL Life Technologies) at 1900 V. The Cy5-labeled amplicons were visualized on the Storm 860 scanner using the software ImageQuant v5.2 (Molecular Dynamics, Sunnyvale, CA). Alleles present in the field samples were compared with those present in the six reference strains, and were numerically designated from 1 to 10, corresponding to their relative electrophoretic mobility positions on the gel (1 = slowest, largest product; 10 = fastest, smallest product). Base pair sizes of msint2 and msint3 PCR products were determined for some of the reference strains (3D7 = 222 and 200; K1 = 190 and 184; PNG1917 = 217 and 147, respectively) (Su X-Z, personal communication). However, in this study, numbers from 1 to 10 were used to designate the alleles [8]. ImageQuant was used to score multiple alleles per locus if the minor fluorescent peaks were >25% of the height of the predominant peak present at each locus. Haplotypes were constructed using the predominant allele observed at each locus.

### Statistical analysis

The population genetics software Arlequin v3.0 [43] was used to compute the heterozygosity (*H*) at each locus (value ± SD), full-length haplotype diversity (value ± SE), and genetic differentiation (*F*_st_), which measures variation between population groups, and LD between loci. *H *and *F*_st _values measure from 0 to 1, where 0 means that there is no difference between individuals or groups, and 1 means that all individuals are unique and population groups are distinct from each other.

## Results

A total of 1287 samples were analysed in this study. Seven of these were placental samples, collected from the Eastern Highlands (n = 1), East Sepik (n = 3), Manus (n = 1), Milne Bay (n = 1), and Morobe (n = 1) provinces between 1982 and 1984. From our more recent field studies, 1280 blood samples were collected from the Dreikikir (n = 31), Liksul (n = 22), and Wosera (n = 1227) regions between 1996 and 2003. The *P. falciparum *infection status of each sample was diagnosed by genotyping *pfcrt *codons 72–76 using LDR-FMA. All samples from the early 1980's, Dreikikir, and Liksul surveys were *P. falciparum*-infected. Of the samples collected from the Wosera, 166 samples from community surveys and 595 samples from health center surveys were *P. falciparum*-infected. Prevalence of genotypically CQS (CVMNK) and CQR (SVMNT and/or CVIET) *P. falciparum *at each sample collection site is summarized in Table 1. Of all samples (n = 1287), 8.9% were infected with CQS parasites, 47.9% with CQR parasites, and 6.9% with mixed infections, while 36.2% were not infected with *P. falciparum*. Interestingly, the CQR-associated *pfcrt *allele CVIET was observed in the Wosera community samples (0.006, n = 1/166) and clinical samples (0.013, n = 8/595) collected after 2001. Previous studies did not observe the CVIET allele in any of the PNG *P. falciparum*-infected samples [7,8].

**Table 1:** Prevalence of *pfcrt *alleles (codons 72–76) in malaria-endemic regions of PNG

	**Early 1980's**^a^	**Post-1995**			
		
		**Community**			**Clinical**
			
**Allele**	n = 7	Liksul^b ^ n = 22	Dreikikir^c ^ n = 31	Wosera^d ^ n = 166	Wosera^e ^ n = 595
CVMNK	0.143	0.045	0.484	0.241	0.097
SVMNT	0.857	0.955	0.516	0.572	0.791
CVIET	0	0	0	0.006^f^	0.013
CVMNK + SVMNT	0	0	0	0.181	0.086
CVMNK + CVIET	0	0	0	0	0.002
SVMNT + CVIET	0	0	0	0	0.012

a, b, c Samples with mixed infections were not included.

a Samples were collected from the Eastern Highlands (n = 1), East Sepik (n = 3), Manus (n = 1), Milne Bay (n = 1), and Morobe (n = 1) provinces of PNG between 1982 and 1984 and were previously reported [8].

b, c Samples were collected in 1996 and were previously reported [7].

d Samples were collected in 1998 and 2002. The 1998 samples were previously reported [7].

e Samples were collected between 2001 and 2003 in local health centers.

f Allele found only in samples taken from the 2002 group.

*Pfcrt*-SVMNT allele carrying samples in both the early 1980's and post-1995 groups were further analysed for SNPs at codons 220, 271, 326, 356, and 371, as well as recently reported SNPs at codons 152 and 163 in exon-3 [30] by LDR-FMA. In this expanded SNP analysis of *pfcrt*-SVMNT allele, S_220_Q_271_D_326_L_356_R_371 _was the predominant haplotype (>0.900). No polymorphism at codons 152 and 163 was observed in any of the samples. There was some variation to the S_220_Q_271_D_326_L_356_R_371 _haplotype in the post-1995 samples, with 220A (0.044, n = 8/181), 271E (0.011, n = 2/176), 326N (0.006, n = 1/149), 356I (0.092, n = 18/195), and 371I (0.059, n = 11/185). Since minor alleles at one or more positions were present as mixed infections, 220_271_326_356_371 minor haplotypes could not be inferred.

**Figure 1:**
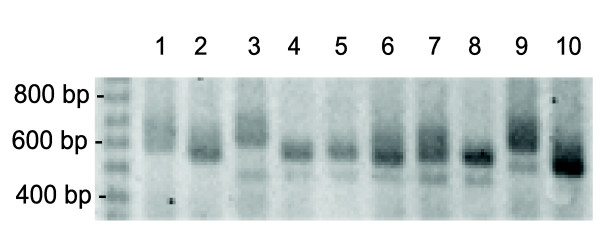
**Agarose gel showing PCR products with size variability**. PCR products for *pfcrt *codons 152–163 genotyping assay showing size variation between samples. Lanes 2, 4, 6, 8, and 10 contain PNG samples collected from the Wosera in 2002. The other lanes contain laboratory-adapted strains, lane 1-Dd2, 3-7G8, 5-PNG1917, 7-PNG1905, and 9-K1.

While performing the amplification of exon-3 region to genotype codons 152 and 163, significant variation in the size of the PCR products was observed when run on a 2% agarose gel (Figure 1). To understand what might be contributing to this size variation, simple sequence repeats in the 3D7 chromosome 7 published sequence were located (GenBank accession number AL844506). An (AT)_23 _repeat was found in intron-2 (nucleotide coordinates, 309138–309183), and an A_40 _repeat was located in intron-3 (nucleotide coordinates, 309439–309478). Due to their intronic locations within the *pfcrt *gene, the MS were named msint2 and msint3 (Additional File 3). Next, to determine if one or both of these MS contributed to the size variation observed for the exon-3 containing PCR products, primers were designed to amplify these sequences using nested strategies. When these products were run on a polyacrylamide gel, it was found that both msint2 and msint3 simple sequence repeats displayed size variations.

In the samples carrying the CQS-associated CVMNK allele, eight different msint2 alleles were observed (predominant alleles #7 [0.266] and #3 [0.241]). Eight different alleles of msint3 were also observed in these samples (predominant alleles #8 [0.395] and #6 [0.296]). In the samples carrying the CQR-associated SVMNT allele, eight msint2 alleles (predominant allele #3 [0.889]) and five msint3 alleles (predominant allele #8 [0.954]) were observed. In the samples carrying the CQR-associated CVIET allele, three msint2 alleles (predominant allele #2 [0.5]) and two msint3 alleles (predominant allele #8 [0.833]) were observed. Allele frequencies for both MS in each subset of samples are presented in Additional File 4.

**Figure 2:**
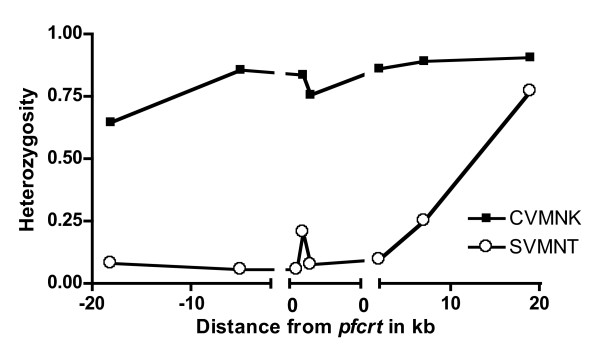
**Heterozygosity of *pfcrt *intronic and flanking MS in *pfcrt*-CVMNK and SVMNT samples**. Graphic representation of heterozygosity values for *pfcrt *intronic and flanking MS in *pfcrt*-CVMNK and SVMNT samples from all study sites. Data for msint1 is available only from *pfcrt*-SVMNT clinical samples collected from the Wosera.

To analyse the levels of diversity at MS loci across the 40 kb region containing *pfcrt*-SVMNT alleles, heterozygosity (*H*) values were calculated for each individual locus. *H *ranged from 0 to 0.781 (Table 2), with the highest value observed at the 2H4 locus (0.71 to 0.781) positioned 19 kb downstream from *pfcrt*. Elevated *H *was also observed at the PfPK2 locus (0.617 to 0.769), which has been previously analysed in broad *P. falciparum *population surveys as a neutral marker under no apparent selection pressure [33,44]. *H *observed at the MS loci in the CQS (*pfcrt*-CVMNK) samples ranged from 0.642 to 0.903. Consistent with observations documenting reduced variation around *P. falciparum *genes associated with antimalarial drug resistance [45], the lowest values were observed for markers in the closest proximity to *pfcrt *(B5M77 to *cg2*; Figure 2). In contrast, elevated values were observed for the marker within *pfcrt *(msint2; Figure 2). Across the 40 kb region where *pfcrt *resides, significant LD (p < 0.05) was observed for eight of the pairwise comparisons (B5M77-B5M47, 2E10-B5M47, B5M77-*pfcrt *codon 371, 2E10-9B12, B5M47-9B12, B5M47-2H4, *cg2*ω-2H4, B5M47-PfPK2) in the CQR samples, and for four of the pairwise comparisons (*pfcrt *codon 220-*pfcrt *codon 356, 2E10-9B12, msint2-2H4, msint2-PfPK2) in the CQS samples. After a correction for multiple comparisons, significant LD was observed for two pairwise comparisons (B5M47-2H4, *cg2*ω-2H4; p < 0.0014) in the CQR samples, and for one pairwise comparison (*pfcrt *codon 220-*pfcrt *codon 356; p < 0.0009) in the CQS samples.

**Table 2:** Heterozygosity (± standard deviation) of MS loci associated with *pfcrt*-SVMNT^a ^allele in post-1995 samples.

**Locus**^c^	**Distance from *pfcrt***	**Liksul ** n = 21	**Dreikikir ** n = 16	**Wosera**^b ^ n = 169
B5M77	-18 kb	0	.342 ± .140	.060 ± .026
2E10	-5 kb	0	.154 ± .126	.049 ± .024
msint1^d^	0 kb	-	-	.051 ± .034^e^
msint2	0 kb	.091 ± .081	.380 ± .134	.196 ± .039
msint3	0 kb	0	.314 ± .138	.056 ± .024
9B12	+2 kb	.181 ± .104	.275 ± .145	.045 ± .030
*cg2*	+7 kb	.450 ± .128	.275 ± .148	.220 ± .042
2H4	+19 kb	.710 ± .060	.781 ± .102	.746 ± .020

PfPK2	---	.617 ± .063	.769 ± .083	.744 ± .019

a 21/22 infections in Liksul, 16/31 in Dreikikir, and 169/761 in Wosera carried the *pfcrt*-SVMNT allele.

b Wosera group contains both clinical and community samples.

c PCR for Liksul samples was 100% for all loci except *cg2 *(.905). Frequency of amplification for each locus for Dreikikir and Wosera, respectively, were B5M77, 0.875, 0.970; 2E10, 0.813, 0.941; 9B12, 0.813, 0.941; *cg2*, 0.813, 0.976; 2H4, 0.875, 0.935; and PfPK2, 0.813, 0.935.

d MS also known as B5M47.

e Value based only on 78 of the 82 clinical samples and no community samples.

*F*_st _values were then calculated for each MS locus between the geographically distinct sample collection sites to test for population subdivision, as might be expected between locations that are separated by distance and other geographic factors that would reduce mixing of parasite populations by limiting human and/or mosquito travel. Results from these analyses (summarized in Table 3) showed similar *F*_st _values for each pairwise comparison. Though locus-by-locus comparisons did show some significant *F*_st _values, overall there is no clear-cut genetic variability among the three locations, suggesting no subdivision among CQR *P. falciparum *populations at these locations.

**Table 3:** *F*_st _values to measure genetic diversity between groups of *pfcrt*-SVMNT samples from post-1995 collections

**Locus**	**Liksul-Dreikikir**	**Liksul-Wosera**	**Dreikikir-Wosera**
B5M77	0.12*	-0.01	0.17*
2E10	0.04	-0.02	0.04
msint2	0.03	-0.01	0.01
msint3	0.07	-0.01	0.12
9B12	-0.04	0.04	0.10
*cg2*	0.00	0.04	-0.01
2H4	0.07*	0.10*	0.08*
PfPK2	0.07*	0.01	0.03

* indicates that the value is significantly different from zero (p < 0.05).

Further, to examine the CQR parasite population structure and distribution among various locations, msint2_msint3 haplotypes in single infection samples were analysed (Table 4). In the early 1980's samples, three haplotypes were observed in the SVMNT samples (n = 6) (haplotype diversity, 0.467 ± 0.426), with the predominant haplotype being 3_8 (0.667). It was of interest to note that the other two haplotypes (8_3 and 3_2) observed in these early samples were not observed in the later samples. Among the post-1995 SVMNT samples (n = 203), 11 haplotypes were observed (haplotype diversity, 0.103 ± 0.140), and the predominant haplotype was again 3_8 (from 0.667 to 0.955). In this intronic haplotype data analysis, it was observed that while the two predominant haplotypes (3_8 and 7_8) were present at all study sites, other low frequency haplotypes were segregated and were unique to each study site (Table 4). In the CVIET samples (n = 6), three haplotypes were present (haplotype diversity, 0.533 ± 0.468), with the predominant haplotype being 2_8 (0.5). Although this intronic haplotype was also observed in association with the SVMNT allele in the Wosera community survey samples (0.011), the other two haplotypes (1_8 [0.333] and 8_7 [0.167]) were unique to the CVIET samples; the 3_8 and 7_8 haplotypes that were predominant among the SVMNT samples were not observed in the CVIET samples. Finally, the intronic MS haplotypes associated with the post-1995 CVMNK samples were examined, and a total of 27 haplotypes in 54 samples were found (haplotype diversity, 0.772 ± 0.519), showing a much higher level of diversity than that observed among the SVMNT samples (Figure 2).

**Table 4:** Intronic haplotype frequencies for *pfcrt*-SVMNT and CVIET samples^a^

	**Early 1980's**	**Post-1995**				
		**Community**			**Clinical**	
			
		Liksul	Dreikikir	Wosera	Wosera	
**Haplotype ** (msint2_msint3)	SVMNT n^b ^= 6	SVMNT n = 22	SVMNT n = 21	SVMNT n = 88	SVMNT n = 82	CVIET n = 6
3_8	0.667	0.955	**0.667**^c^	**0.898**	**0.951**	0
7_8	0	0.045	**0.095**	**0.057**	**0.037**	0
8_3	0.167	0	0	**0**	**0**	0
3_2	0.167	0	0	0	0	0
7_4	0	0	**0.048**	**0**	0	0
3_3	0	0	0.048	0	0	0
3_6	0	0	0.048	**0**	**0**	0
6_6	0	0	**0.048**	0	0	0
10_8	0	0	0.048	0	0	0
8_8	0	0	0	**0.023**	**0**	0
9_8	0	0	0	0.011	0	0
2_8	0	0	0	0.011	0	0.500
4_8	0	0	0	0	**0.012**	0
1_8	0	0	0	0	0	0.333
8_7	0	0	0	0	0	0.167

a Total number of samples 215: early 1980's (6), Liksul (21), Dreikikir (16), Wosera community samples (85), Wosera clinical samples (81 SVMNT, 6 CVIET).

b "n" indicates the number of haplotype infections present, which may or may not be equal to the total number of samples due to mixed haplotypes in some samples. Overall, 225 haplotypes were found in 215 samples.

c Frequencies in bold indicate that the haplotype was also observed in the CQS samples from that region.

## Discussion

Following the discovery of the association between *pfcrt *K76T and *P. falciparum *CQR [11], Wootton et al. evaluated a number of MS loci flanking *pfcrt *(± 100 kb) of 87 laboratory-adapted strains collected from malarious regions around the world to investigate the impact of CQ selection pressure on the genetic diversity in this region of the genome [35]. Results of this study comparing CQS and CQR-associated alleles showed significant reduction in MS allelic diversity, increased LD, and uniform haplotypes for the markers in the closest proximity to the *pfcrt *CQR allele [35]. They suggested that CQ was responsible for selection-driven "sweeps", homogenising genetic diversity at polymorphic sites in close physical linkage with CQR-associated alleles (hitchhiking) [35]. Similar observations have been made using MS flanking the alleles associated with pyrimethamine resistance at the dihydrofolate reductase gene (chromosome 4) [34,46-49] and quinine resistance at *pfnhe-1*, encoding a putative Na(+)/H(+) exchanger (chromosome 13) [50]. A more recent study of MS flanking *pfcrt*, including some of the same samples analysed here, used these approaches to describe population subdivision between the early 1980's and late 1990's CQR *P. falciparum *strains in PNG [8].

With these observations in mind, it was expected that very low or no heterozygosity would be observed, similar to that reported by Wootton et al. for msint1 (B5M47), at msint2 and msint3 in CQR *P. falciparum *strains. Interestingly, this and a previous study [31] found higher levels of MS variability within the introns of the *pfcrt *gene than the variability at MS loci flanking *pfcrt *(Figure 2; msint2 vs. 2E10 or 9B12, [0.201 vs. 0.051 or 0.094]). As the flanking and intronic MS sites are similar in their length and nucleotide content, two of the features associated with MS variability, it is not clear what genetic factors contribute to these higher levels of variability in *pfcrt *introns 2 and 3.

Intronic polymorphisms may provide important insights into maintenance of the *pfcrt *gene sequence. In addition to the MS length polymorphism reported now in *pfcrt *msint2, msint3 and msint4, a review of genomic sequences currently available at GenBank for *P. falciparum *strains 3D7 (GenBank accession number AL844506) and Dd2 (GenBank accession number AF030694) showed that simple sequence repeats were present within all *pfcrt *introns. A comparison between 3D7 and Dd2 genomic sequences has shown additional length polymorphism for simple sequence repeats in introns 7 (T_14 _vs. T_13_), 9 (AT_18 _vs. AT_15_), 10 (T_14 _vs. T_15_), and two in intron-11 (T_18 _vs. T_23 _and T_31 _vs. T_19_). The highly AT-rich and polymorphic characteristics of many intronic repeats has been described for a number of *P. falciparum *genes [51]. The high level of intronic diversity has been suggested to result from slippage of DNA polymerase [32,33]. Alternatively, repetitive AT-rich sequences that are palindromic have the potential to form intrastrand base-pairing, resulting in hairpins that are susceptible to breakage followed by meiotic recombination [52]. Events of this nature could contribute to gene conversion where polymorphic exons are swapped between parental alleles in the formation of new progeny alleles [53,54]. Further evaluation of *pfcrt *in laboratory adapted strains and natural *P. falciparum *isolates may reveal sequence-based relationships supportive of the hypothesis that these loci promote recombination/gene conversion within this genomic region [55].

In addition to evaluating *pfcrt *intronic MS, this study provides the first evidence of the CQR-associated CVIET allele in PNG. While CVIET is the predominant CQR-associated *pfcrt *allele in many parts of Southeast Asia [11,35], earlier studies in PNG [7,8] did not find this allele in any of the samples. Since the allele is found in nearby Indonesian regions [56,57], it is likely that this allele has been imported into PNG. When intronic MS loci in *pfcrt*-SVMNT and *pfcrt*-CVIET samples were compared, significant genetic differences were found between the two groups. The predominant msint2_msint3 haplotype 3_8 associated with *pfcrt*-SVMNT was not seen in *pfcrt*-CVIET samples. The haplotype diversity was significantly higher in the *pfcrt*-CVIET samples (0.533 ± 0.468) than in the *pfcrt*-SVMNT samples (0.103 ± 0.14). Further, when flanking MS haplotype (from B5M77 to 2H4) diversity was compared between the two groups of samples from the Wosera, the extended haplotype diversity was also significantly higher in the *pfcrt*-CVIET samples (0.7 ± 0.728) than in the *pfcrt*-SVMNT samples (0.06 ± 0.077). These analyses rule out the possibility that the CVIET allele in PNG has arisen on the genetic background of more prevalent SVMNT allele.

Analysis of the diversity at intronic MS provides an "inside look" at the genetic background of *pfcrt *from an evolutionary perspective. Among all *pfcrt*-SVMNT samples, the predominant msint2 allele #3 and msint3 allele #8 (msint2_msint3 haplotype 3_8) in the early 1980's samples as well as in the post-1995 samples from three different malaria-holoendemic regions were observed. In all post-1995 samples, one other predominant haplotype 7_8 as well as lower frequency haplotypes, unique to each location, were observed. These data indicate that during the early spread of CQR, a predominant msint2_msint3 haplotype swept across PNG under CQ selection, and now has independently accumulated diversity over time in different regions of PNG. These results are consistent with the results of a previous study [8], where significantly higher diversity was observed at the MS loci flanking *pfcrt*-SVMNT in the late 1990's samples than in the early 1980's samples. This suggests that although SVMNT remains the predominant CQR-associated *pfcrt *allele in PNG, its genetic background has accumulated significant diversity even under drug selection pressure.

Similar to the observations shown here, Vinayak et al. [31] found that a single major haplotype between *pfcrt *msint2_msint4 (AT_TA repeats; frequencies 0.222–0.879) was distributed across their six Indian sample collection sites. The frequencies of the minor haplotypes ranged from 0.02–0.244; 12 of the 32 minor haplotypes were distributed among sites separated by thousands of kilometers. In PNG, the major *pfcrt *msint2_msint3 haplotype (3_8) was present at all three sample collection sites at frequencies ranging from 0.667–0.955, while the 9 minor haplotypes were unique to each of the sites, and ranged in frequency from 0.011–0.048 (Table 4). Vinayak et al. [31] did not evaluate the polymorphism in msint3. Interestingly, when some of the same Indian samples were analysed for msint2_msint3 haplotypes, it was found that the same haplotype predominant in PNG (3_8) was also present in Indian samples at a frequency of 0.917 (n = 22/24). This observation suggests a relationship between PNG and Indian CQR *P. falciparum *strains that should be further evaluated. Finally, although Vinayak et al. [31] observed overall reduced msint2_msint4 haplotype diversity in lower malaria transmission areas, in PNG the lowest msint2_msint3 diversity was observed in the Liksul region (Madang area), which is known to experience holoendemic malaria transmission. As malaria ecology is known to be very different between PNG and India, it is possible that local factors influencing *P. falciparum *transmission dynamics contribute to some of the overall differences in frequency and distribution of intronic MS polymorphism between these two studies.

Recently, Ariey et al. used msint4 to look at the ancestry of CQR parasites at 16 survey sites throughout Africa [58]. By analysing the MS distribution in parasites carrying sensitive and resistant alleles, they reported a significant difference in the level of variability with 17 MS alleles present in the sensitive parasites vs. two MS alleles present in the resistant parasites [58]. Because 123 of the 125 resistant parasites from wide ranging survey sites carried the same MS allele, Ariey et al. concluded that Africa was invaded by a single CQR *P. falciparum *strain [58]. While it is clear from Ariey et al. [58] that a single intron-4 MS allele predominates among African CQR *P. falciparum*, results shown here and by Vinayak et al. [31] provide evidence that multiple intronic MS within *pfcrt *are polymorphic, and they have differing levels of variability. Therefore, to fully understand the ancestry, dispersal, and population genetics of CQR *P. falciparum *in Africa, or throughout the world, more complete surveillance of *pfcrt *intronic MS should be conducted. It would also be interesting to examine earlier sample sets to determine if parasites with a single *pfcrt *intronic MS haplotype invaded Africa, or if multiple haplotypes occurred and only one became predominant. Of further interest, given the recent reports that removal of CQ pressure in Africa is associated with a return of the wild-type *pfcrt *allele carrying strains and CQ sensitivity [59], analysis of polymorphisms in both intronic and flanking MS may provide useful insight regarding drug sensitivity distribution patterns.

## Conclusion

In conclusion, highly polymorphic MS loci have been identified within the *pfcrt *gene. These new markers were used to reassess the relationships among *P. falciparum *in blood samples obtained from the early 1980's through 2003 from malaria-endemic sites in Madang and East Sepik Provinces of PNG. These results suggest that a single major haplotype associated with CQR has achieved widespread distribution in PNG, and that this sequence continues to generate new polymorphism within localized regions. Although the analysis shown here has focused largely on MS polymorphism associated with the common *pfcrt*-SVMNT allele, for the first time, the identification of the *pfcrt*-CVIET allele in PNG is reported. These CVIET parasites contain intronic MS haplotypes that are not present in the SVMNT parasites, suggesting that the CVIET parasites may have been imported from neighboring geographic regions. By using *pfcrt *intronic MS as CQ susceptibility markers, the continuing evolution of *pfcrt *can be monitored, the geographic and temporal patterns of CQS and CQR parasite populations can be further analysed, and relative fitness of CQS and CQR *P. falciparum *strains can be evaluated.

## Authors' contributions

JTD and RKM performed the molecular typing of samples, data analysis, and interpretation, and wrote the manuscript. PM, IM, and JR collected and organised the PNG samples as well as developed the experimental design. YDS provided the Indian field samples and contributed to the experimental design. MS helped in data analysis and was involved in drafting the manuscript. PAZ was instrumental in the experimental design, data analysis, completion of the manuscript, and raising funds for this study.

## Supplementary Material

Additional File 1 Primers and PCR amplification conditions for *pfcrt *loci. Click here for file

Additional File 2 Ligase detection reaction primers for genotyping *pfcrt *codons. Click here for file

Additional File 3 Schematic of PCR primers used to characterise *pfcrt *intronic microsatellites. Click here for file

Additional File 4 Allele frequencies of intron-2 and intron-3 microsatellites in malaria-endemic regions of Papua New Guinea. Click here for file
