# Supplementary material for: Microsatellite polymorphism within pfcrt provides evidence of continuing evolution of chloroquine-resistant alleles in Papua New Guinea
Source: Malar J. 2007 Mar 21;6:34. doi: 10.1186/1475-2875-6-34 (PMC1838424; doi:10.1186/1475-2875-6-34)
Supplement: Additional File 1 — Primers and PCR amplification conditions for pfcrt loci. [file 1475-2875-6-34-S1.doc]

| **Additional File 1.** | |  |
| --- | --- | --- |
| **Primers and PCR amplification conditions for *pfcrt* loci** | |  |
| *pfcrt* loci primers | Primer sequences^a^ | Amplification conditions |
| Nest-1 | Up(308515) 5’- CATATAACAAAATGAAATTCGCAA -3' | 94^o^C 3 min (1x), 94^o^C 30 sec, 56^o^C 30 sec, 60^o^C 1 min (35x), 60^o^C 3 min (1x) |
| msint1^b^ | Dn(308823) 5’-GACAAGAACCTCCACCTAAACG -3' |
|  |  |
| Nest-2 | Up(308572) 5’- ATGACGAGCGTTATAGAGAA -3' | 94^o^C 3 min (1x), 94^o^C 30 sec, 56^o^C 30 sec, 60^o^C 1 min (25x), 60^o^C 3 min (1x) |
| msint1^b^ | Dn(308734) Cy5-5’- ATACATATGGGTATACATATA -3' |
|  |  |
| Nest-1 | Up(309031) 5’- CCTTATTTGGAAATAAAAAGGG -3’ | 94^o^C 3 min (1x), 94^o^C 30 sec, 56^o^C 30 sec, 60^o^C 1 min (35x), 60^o^C 3 min (1x) |
| msint2 and msint3 | Dn(309645) 5’- CTACAAGAGCTATTGTTACAAC -3’ |
| and codons 152-163 |  |
| Nest-2 | Up(309097) 5’- ATAAGAGAATCTATTCCACCTACC -3' | 94^o^C 3 min (1x), 94^o^C 30 sec, 56^o^C 30 sec, 60^o^C 1 min (25x), 60^o^C 3 min (1x) |
| codons 152-163 | Dn(309613) 5’- GCTCCGAGATAATTGTATAAGTGA -3' |
|  |  |
| Nest-2 | Up(309097) 5’- ATAAGAGAATCTATTCCACCTACC -3’ | 94^o^C 3 min (1x), 94^o^C 30 sec, 56^o^C 30 sec, 60^o^C 1 min (25x), 60^o^C 3 min (1x) |
| msint2 | Dn(309319) Cy5-5’- TATGAAGGCCAAAATGACTG -3’ |
|  |  |
| Nest-2 | Up(309408) 5’- GATATAGGTAAGTATACTATTTT-3’ | Same as above |
| msint3 | Dn(309613) Cy5-5’- GCTCCGAGATAATTGTATAAGTGA -3’ |  |
| [a Position of primer shown in parenthesis is based on GenBank accession number AL844506.](http://www.ncbi.nlm.nih.gov/entrez/viewer.fcgi?db=nucleotide&val=23498713) | |  |
| b MS locus also known as B5M47 | |  |
