# Supplementary material for: Microsatellite polymorphism within pfcrt provides evidence of continuing evolution of chloroquine-resistant alleles in Papua New Guinea
Source: Malar J. 2007 Mar 21;6:34. doi: 10.1186/1475-2875-6-34 (PMC1838424; doi:10.1186/1475-2875-6-34)
Supplement: Additional File 2 — Ligase detection reaction primers for genotyping pfcrt codons. [file 1475-2875-6-34-S2.doc]

| **Additional File 2.** | |  |
| --- | --- | --- |
| **Ligase Detection Reaction primers for genotyping *pfcrt* codons** | |  |
| Codon/s - allele | Primer Sequence^a^ | FlexMap^TM^ |
|  |  | microsphere |
| 72-76 SVMNT | 5'-AATCCTTTCTTTAATCTCAAATCAatttaagtgtaagtgtaatgaatac-3' | 21 |
| 72-76 CVIET | 5'-TCATAATCTCAACAATCTTTCTTTatttaagtgtatgtgtaattgaaac-3' | 68 |
| 72-76 CVMNK | 5'-AATCTACAAATCCAATAATCTCATatttaagtgtatgtgtaatgaataa-3' | 60 |
| 72-76 common | 5'/Phos/aatttttgctaaaagaactttaaac3' Biotin |  |
|  |  |  |
| 152 A | 5'-TCAAAATCTCAAATACTCAAATCAtcataggtcttacaagaactg-3' | 18 |
| 152 T | 5'-TCATCAATCAATCTTTTTCACTTTtcataggtcttacaagaacta-3' | 59 |
| 152 common | 5'/Phos/ctggaaatatccaatcatttg3' Biotin |  |
|  |  |  |
| 163 S | 5'-TTACCTTTATACCTTTCTTTTTACaatcatttgttcttcaattaagt-3' | 30 |
| 163 R | 5'-TACACTTTCTTTCTTTCTTTCTTTaatcatttgttcttcaattaagg-3' | 12 |
| 163 common | 5'/Phos/attcctattaatatgttcttctg3' Biotin |  |
|  |  |  |
| 220 A | 5'-TCATTTACCAATCTTTCTTTATACtttaatcttgtcttaattagtgcc-3' | 44 |
| 220 S | 5'-CTTTTCATCAATAATCTTACCTTTtttaatcttgtcttaattagttcc-3' | 65 |
| 220 common | 5'/Phos/ttaattgtaagaaaacaaaatatata-3' Biotin |  |
|  |  |  |
| 271 Q | 5'-CTACTATACATCTTACTATACTTTtacacccttccatttttaaaacaa-3' | 14 |
| 271 E | 5'-AAACTAACATCAATACTTACATCAtacacccttccatttttaaaagaa-3' | 87 |
| 271 common | 5'/Phos/cgtaagaattaattaggaaagaaaa-3' Biotin |  |
|  |  |  |
| 326 S | 5'-CTTTTCATCTTTTCATCTTTCAATttcgcattgttttgcttctttagc-3' | 37 |
| 326 D | 5'-CTTTAATCCTTTATCACTTTATCAttcgcattgttttgcttctttgac-3' | 17 |
| 326 N | 5'-TTACCTTTATACCTTTCTTTTTACttcgcattgttttgcttccttaac | 30 |
| 326 common | 5'/Phos/atttgtgataatttaataaccagct-3' Biotin |  |
|  |  |  |
| 356 I | 5'-TCAATCATAATCTCATAATCCAATgttgtatacaagctccagcaata-3' | 62 |
| 356 T | 5'-CTACAAACAAACAAACATTATCAAgttgtatacaagctccagcaaca-3' | 28 |
| 356 L | 5'-CAATTTCATCATTCATTCATTTCAgttgtatacaagctccagcatta | 35 |
| 356 common | 5'/Phos/gcaattgcttattactttaaattct-3' Biotin |  |
|  |  |  |
| 371 R | 5'-AATCCTTTTTACTCAATTCAATCAattttatagggtgatgttgtaaga-3' | 22 |
| 371 I | 5'-TACACTTTCTTTCTTTCTTTCTTTattttatagggtgatgttgtaata-3' | 12 |
| 371 common | 5'/Phos/gaaccaagattattagatttcgtaa-3' Biotin |  |
| [a Primers were designed using 3D7 chromosome 7 sequence (GenBank accession number AL844506). Nucleotides in upper case](http://www.ncbi.nlm.nih.gov/entrez/viewer.fcgi?db=nucleotide&val=23498713) letters (24 bases) represent the “TAG” sequence added to the 5’ end of each allele-specific LDR primer. | | |
