# Supplementary material for: Microsatellite polymorphism within pfcrt provides evidence of continuing evolution of chloroquine-resistant alleles in Papua New Guinea
Source: Malar J. 2007 Mar 21;6:34. doi: 10.1186/1475-2875-6-34 (PMC1838424; doi:10.1186/1475-2875-6-34)
Supplement: Additional File 4 — Allele frequencies of intron-2 and intron-3 microsatellites in malaria-endemic regions of Papua New Guinea. [file 1475-2875-6-34-S4.doc]

| **Additional File 4.** | | |  |  |  |  |  |  |  |  |  |  |
| --- | --- | --- | --- | --- | --- | --- | --- | --- | --- | --- | --- | --- |
| **Allele frequencies of *pfcrt* intron-2 and 3 microsatellites in malaria-endemic regions of Papua New Guinea** | | | | | | | | | | | |  |
|  | **Early 1980's** | | **Post-1995** | | | | | | | | | |
| **MS** |  |  | **Community** | | | | | |  | **Clinical** | | |
| **allele** |  |  | Liksul | | Dreikikir | | Wosera | |  | Wosera | | |
|  | CVMNK | SVMNT | CVMNK | SVMNT | CVMNK | SVMNT | CVMNK | SVMNT |  | CVMNK | SVMNT | CVIET |
| **msint2** | n = 2 | n = 6 | n = 1 | n = 22 | n = 18 | n = 19 | n = 46 | n = 95 |  | n = 12 | n = 84 | n = 6 |
| 1 | 0 | 0 | 0 | 0 | 0 | 0 | 0 | 0 |  | 0 | 0 | 0.333 |
| 2 | 0 | 0 | 0 | 0 | 0 | 0 | 0 | 0.011 |  | 0 | 0 | 0.500 |
| 3 | 0.500 | 0.833 | 0 | 0.955 | 0.278 | 0.789 | 0.239 | 0.853 |  | 0.167 | 0.940 | 0 |
| 4 | 0 | 0 | 0 | 0 | 0.056 | 0 | 0.043 | 0 |  | 0.417 | 0.012 | 0 |
| 5 | 0 | 0 | 0 | 0 | 0.111 | 0 | 0.087 | 0 |  | 0 | 0 | 0 |
| 6 | 0 | 0 | 0 | 0 | 0.222 | 0.053 | 0.043 | 0 |  | 0 | 0 | 0 |
| 7 | 0.500 | 0 | 1.000 | 0.045 | 0.278 | 0.105 | 0.283 | 0.105 |  | 0.083 | 0.048 | 0 |
| 8 | 0 | 0.167 | 0 | 0 | 0 | 0 | 0.217 | 0.021 |  | 0.333 | 0 | 0.167 |
| 9 | 0 | 0 | 0 | 0 | 0 | 0 | 0.065 | 0.011 |  | 0 | 0 | 0 |
| 10 | 0 | 0 | 0 | 0 | 0.056 | 0.053 | 0.022 | 0 |  | 0 | 0 | 0 |
| **msint3** | n = 2 | n = 6 | n = 1 | n = 21 | n = 20 | n = 18 | n = 47 | n = 91 |  | n = 11 | n = 83 | n = 6 |
| 1 | 0 | 0 | 0 | 0 | 0 | 0 | 0 | 0 |  | 0 | 0 | 0 |
| 2 | 0 | 0.167 | 0 | 0 | 0 | 0 | 0 | 0 |  | 0 | 0 | 0 |
| 3 | 0 | 0.167 | 0 | 0 | 0 | 0.056 | 0.021 | 0 |  | 0.091 | 0 | 0 |
| 4 | 0 | 0 | 0 | 0 | 0.100 | 0.056 | 0.021 | 0 |  | 0.091 | 0 | 0 |
| 5 | 0 | 0 | 0 | 0 | 0.100 | 0 | 0.043 | 0 |  | 0.273 | 0 | 0 |
| 6 | 0.500 | 0 | 1.000 | 0 | 0.250 | 0.056 | 0.319 | 0.044 |  | 0.182 | 0.012 | 0 |
| 7 | 0 | 0 | 0 | 0 | 0 | 0 | 0.106 | 0 |  | 0.091 | 0 | 0.167 |
| 8 | 0.500 | 0.667 | 0 | 1.000 | 0.500 | 0.833 | 0.383 | 0.956 |  | 0.273 | 0.988 | 0.833 |
| 9 | 0 | 0 | 0 | 0 | 0 | 0 | 0.085 | 0 |  | 0 | 0 | 0 |
| 10 | 0 | 0 | 0 | 0 | 0.050 | 0 | 0.021 | 0 |  | 0 | 0 | 0 |
| "n" indicates the number of allele infections present, which may or may not be equal to the total number of samples due to mixed alleles in some samples. Total number of single infection (either CQS or CQR) samples 281: early 1980’s (1, 6), Liksul (1, 21), Dreikikir (15, 16), Wosera (community 36, 87; clinical 10, 82 [SVMNT] and 6 [CVIET]). | | | | | | | | | | | | |
